# Niosome as a drug carrier for topical delivery of N-acetyl glucosamine

**Published:** 2010

**Authors:** M.A. Shatalebi, S.A. Mostafavi, A. Moghaddas

**Affiliations:** Department of Pharmaceutics and Isfahan Pharmaceutical Sciences Research Center, School of Pharmacy and Pharmaceutical Sciences, Isfahan University of Medical Sciences, Isfahan, I.R.Iran

**Keywords:** Noisome, N-Acetyl Glucosamine, Topical delivery, HPLC

## Abstract

Niosomes are non-ionic surfactant vesicles that have potential applications in the delivery of hydrophilic and hydrophobic drugs. The topical form of N-acetyl glucosamine (NAG) recently has been considered in the treatment of hyperpigmentation disorders due to its inhibitory effect on thyrosinase enzymes in melanocytes. To improve NAG penetration into the skin we formulated the drug in niosomes and investigated its flux across excised rat skin using Franz diffusion cells. The drug assay was performed by a novel and specific high performance liquid chromatography method. Niosomal vesicles were further characterized by optical and scanning electron microscopy and particle size analysis. Niosomes prepared with Span 40 produced a drug encapsulation of about 50%. The vesicle size was markedly dependent on the composition of the niosome formulations and was in range of 500-4500 nm (Span 80 < Span 60 < Span 40 niosomes). Span 40-niosomes provided a higher NAG flux across the skin than Span 60- and Span 80-nisomes. All formulations significantly improved the extent of drug assessed to be localized in the skin (P< 0.05), as compared to NAG hydroalcoholic (HA) solution. Our study demonstrated the potential of niosomes for improved NAG localization in the skin, as needed in hyperpigmentation disorders.

## INTRODUCTION

Colloidal particulate carriers such as liposomes(1) or niosomes(2) as drug delivery systems have distinct advantages over conventional dosage forms. These carriers can act as drug reservoirs, and have been utilized to direct drug at the target organ/tissue. Many active compounds have limited aqueous solubility, so there is a great need for delivery systems suitable for both hydrophobic and hydrophilic drugs. Liposomes and niosomes can carry hydrophilic drugs by encapsulation or hydrophobic drugs by partitioning of these drugs into hydrophobic domains(3). Liposomes have been used as drug carriers to reduce toxicity, increase drug stability, enhance therapeutic effects, prolong circulation time and promote uptake of the entrapped drugs into the target site(4–6). However, they suffer some disadvantages like the lack of stability due to hydrolysis of phospholipid molecules and high manufacturing costs of phospholipids(7).

Alternatively, non-ionic surfactant vesicles or niosomes whose structure and properties are similar to liposomes have been developed. Niosomes are microscopic lamellar structures formed on admixture of non-ionic surfactant of the alkyl or dialkyl polyglycerol ether and cholesterol with subsequent hydration in aqueous media(8) and are capable of entrapping hydrophilic and hydrophobic solutes(9). Technically, niosomes are promising drug carriers because they posses greater stability, less purity variability, and lower cost(10). Niosomes also have the advantages of simple method of production for the routine laboratory use and the possibility of large scale production, when needed.

**Fig. 1:**
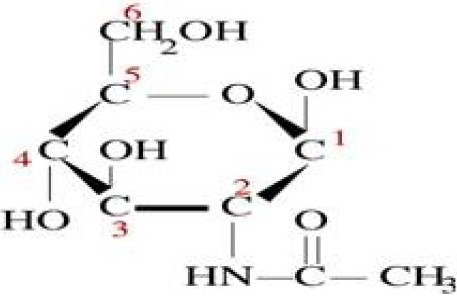
N-acetyl glucosamine chemical structure.

N-acetyl glucosamine (NAG) is chemically an amide between glucosamine and acetic acid. It has a molecular formula of C_6_H_15_NO_6_ (Fig. 1), a molar mass of 221/21 g/mol and, is significantly available in several biological systems.

NAG is best known for its role as a precursor to hyaluronic acid. This polymer serves important structural and hydration roles in extracellular matrix in tissues such as joints and skin both in the epidermis and the dermis(11–13). Glucosamine itself has also been reported to be effective in reducing production of melanin in melanocytes in culture(14–20). The mechanism identified is inhibition of glycosylation of tyrosinase, a limiting enzyme central to formation of melanin. In human pigmentation, glycosylation is a required process in the conversion of inactive protyrosinase to active tyrosinase. This mechanism certainly has potential to reduce melanin production in human skin(21,22). Although glucosamine is of interest for clinical appli-cation, its instability may impede benefiting from its potentials for topical use(23). To the contrary, NAG which is a modest skin lightening agent favors higher stability and can be instead utilized in topical dosage forms. Therefore, we aimed to formulate NAG in niosomes to improve its stability and enhance its penetration into the viable skin. The influence of formulation components on the vesicle size, drug encapsulation efficiency, the drug release from the vesicles and its penetration into the skin were therefore evaluated in a systematic manner.

## MATERIALS AND METHODS

### Materials

NAG was generously donated from Exquim Pharmaceuticals Ind (Spain). Span 40, 60, 80, acetonitrile HPLC grade, chloroform, ethanol and methanol analytical grade were purchased from Merck Company (Darmstadt, Germany). Dicetyl phosphate (DCP) was obtained from Winlab Company (Maidenhead, Germany). Cholesterol (Chol) was obtained from Dow Chemical Company (Midland, MI, USA).

### Assay of NAG by HPLC

#### Chromatographic conditions

Chromatography was performed on a covalent bonded polyamine column (NH2-Phenyl group) which is specifically optimized for the separation of mono- and oligosaccharides. The chromatographic system consisted of the following components: two Jusko PV- 980 pumps, a Millipore instrument degasser, a Jusko dual absorbance variable wavelength detector and HPLC processing software (Borwin^®^). Chromatographic analyses were performed at 22 ± 1 °C; using an -NH2-Phenyl group (250 mm×4 mm i.d., 5 µm particle size). The mobile phase consisted of acetonitrile:water (70:30 v/v) and was pumped at a flow rate of 1 ml/min. The analytes were detected at 205 nm.

#### Sample preparation for assay validation

A 10 mg/ml stock solution of NAG was prepared by dissolving NAG in ethanol 96% and stored in 4 ml amber glass vials at -20 °C until use. Five standard solutions were prepared daily by dilution of stock solutions with ethanol 96% to give NAG concentrations in the range of 1-10 µg/ml to study linearity, intra-day and inter-day variability of HPLC responses. Precision and accuracy were evaluated by performing five consecutive injections of five series of standard solutions during five consecutive days. Prior to injecting the solutions, the column was equilibrated for at least 30 min with the mobile phase flowing through the system. Each standard solution was injected five times during five days, and acceptable results were analyzed statistically. The performance of the HPLC assay was assessed using the following parameters: suitability, linearity, limit of quantitation (LOQ), limit of detection (LOD), precision and accuracy.

### Preparation of niosomes

Niosomes containing NAG were prepared by the film hydration technique with some modifications(3). Formulations were comprised of surfactant (Span 40, Span 60, or Span 80):chol:DCP (X:3.35:1 mole ratio), where the mole ratio of Spans (X) used were as 2.6 (low), 2.9 (medium), or 3.2 (high). To prepare niosomes, an appropriate amount of the surfactant together with 67 µmol of cholesterol and 20 µmol of DCP were dissolved in 30 ml chloroform: methanol (2:1 v/v) in a 250 ml round bottom flask. The solvent was evaporated using a rotary evaporator at 60°C and 70 rpm until a thin layer of lipid film was formed on the inner wall of the flask. The film was subsequently hydrated with 12 ml water containing 2.72 mg NAG and shaken in a water bath at 80 °C for 1 h. The suspension obtained was homogenized for 2 min in an ultrasonic bath. To complete the hydration of the film, the vials were kept at room temperature for 24 h.

### Experimental design

A two-factor (one numeral factor, one categorical factor) linear response surface design at three-level with two center points was adopted in the present study to investigate the effects of the surfactant type (Span 40, Span 60, and Span 80) and surfactant relative concentration (as low, medium, and high) as independent variables on the physical properties, drug release, and drug penetration behaviors of NAG-niosomes, as dependent variables (responses). Statistical treatment of experimental data was carried out using Design Expert^®^, version 7.1.5. The total number of formulations generated by the software was 18, two from each combination. The description of formulations is shown in Table 1 and 2. All studies also were performed on the optimal formulation, which was suggested by the aforementioned program, based on the early particle size, the encapsulation efficiency and the permeability results analysis.

**Table 1:** Independent formulation variables and their levels used in two factorial (one numeral, one categorical) linear experimental design formulation

Variables	Levels			
Surfactant Type	Span 40	Span 60	Span 80
Surfactant relative concentration	Low (-1)	Medium (0)	High (+1)

Mole ratio: Low (2.6), Medium (2.9) and High (3.2)

**Table 2:** The compositions of different niosome formulations.

Formulation Code	Lipid Composition	Mole ratio
Span 40-1	Span 40:Chol:DCP	2.6:3.35:1
Span 40-2	Span 40:Chol:DCP	2.9:3.35:1
Span 40-3	Span 40:Chol:DCP	3.2:3.35:1
Optimum	Span 40:Chol:DCP	3.1:3.35:1
Span 60-1 (optimum)	Span 60:Chol:DCP	2.6:3.35:1
Span 60-2	Span 60:Chol:DCP	2.9:3.35:1
Span 60-3	Span 60:Chol:DCP	3.2:3.35:1
Span 80-1	Span 80:Chol:DCP	2.6:3.35:1
Span 80-2	Span 80:Chol:DCP	2.9:3.35:1
Span 80-3	Span 80:Chol:DCP	3.2:3.35:1

Chol: Cholesterol, DCP: Dicetyl phosphate, in all formulation NAG was 0.0122 mole or 2.75 mg.

### NAG encapsulation efficiency

The niosome suspension was centrifuged at 14000 rpm at 4°C for 20 min. The clear supernatant was removed by pipeting, and the precipitate was washed five times with deionized water to remove all unentrapped NAG. The precipitate was then dissolved in ethanol 96% by the aid of sonication for 20 min. NAG content of noisomes was measured by HPLC, as described before. The encapsulation efficiency (EE%) was calculated by the ratio of NAG amount trapped in pellets to the initial amount of NAG used to prepare the niosomes, assumed as one-hundred percent.

### Microscopy of niosomes

One drop of niosome suspension was mounted on a slide, covered with cover slide and studied under light microscopy (Leitz, Hmlux3, Grmany). In another experiment, one drop of niosomes was mounted on an aluminum stub using double-sided adhesive carbon tape. The vesicles were then sputtercoated with gold palladium (Au/Pd) using a vacuum evaporator (Edwards) and examined using a scanning electron microscope (JSM 5510, Jeol Ltd, Tokyo, Japan) equipped with a digital camera, at 20 KV accelerating voltage.

### Determination of particle size, particle size distribution and zeta potential

The particle size, particle size distribution and zeta potential of the NAG noisomes were determined by Malvern particle size analyzer (ZEN 3600, England).

### In vitro release study

A set of Franz diffusion cells was used to study the release of NAG from niosomes. The diameter of the Franz cells used was 3.6 cm corresponding to an effective permeable area of 3cm. The receptor chamber filled with 38 ml ethanol (96%). The receptor medium was stirred by a magnetic bar and its temperature was kept at 37 °C by circulating water through a jacket surrounding the cell body throughout the experiment.

A cellulose acetate membrane which was already kept in 96% ethanol for 24 h was clamped between two chambers. Three ml of the noisome suspension or HA drug solution was placed in the donor cell. Subsequently, 3 ml samples were collected at 0.5, 1, 2, 4, 6, 8, 12 h from the receptor cell. The same volume of fresh solvent was used to replace the sample after each collection. The collected samples were subjected to HPLC analysis for determination of NAG and the percent of drug release was calculated by taking into account the frequent dilution occurred throughout the experiment.

### Rat skin permeation study

The male Sprague-Dawley rat of three months age (Pasteur Institute of Iran, Tehran) was used to study the permeation of NAG through skin. The rats were sacrificed; their hairs were clipped away with scissors and their skins were subjected to depilatory for 10 min which was further wiped off with cotton. The experiments were performed with permission of the Animal Ethics Committee of Isfahan University of Medical Sciences. An incision was made on the flank of the animal and the skin was separated from the underlying connective tissue using scalpel. The fat remaining on the skin was trimmed away using a razor blade. The residual hypodermal fat was rubbed off by a piece of isopropyl alcoholimpregnated cotton. The skin so prepared was kept between the glass plates at 4 °C and used within two days.

On the day of experiment, the rat skin was transferred to the normal saline solution, where it was kept for about 1 h. The skin was cut, placed between two Franz diffusion cells, and clamped. Three ml NAG niosome suspension and HA solution were placed on the epidermal side of the skin in the donor cell. The receptor cell was filled with ethanol 96% and the experiment was carried out under the same conditions as *in vitro* release study.

### Assessment of NAG permeability and flux from the rat skin

The cumulative quantities of NAG (M) permeated through the surface unit of the membrane (S) were plotted against time. The flux (Jss, µg/cm^2^.h) was obtained from Eq. 1 and the permeability coefficient (P, cm/h) was determined from the slope of the plot in the steady state (Eq. 2) (25), assuming the drug concentration in the donor cell (C_d_, µg/ml) was constant throughout the course of study.

Eq. 1 $$M/S=J_{SS}t$$

Eq. 2 $$J_{SS}=P.C_{d}$$

At the end of experiments (at 24 h), the surface of the skin was thoroughly washed (5X) with ethanol 96%, sonicated for 20 min, and subjected to the HPLC, for determination of percent drug remained on the skin. Subsequently, the percent of drug penetrated into (and retained and localized in) the skin was estimated by the subtraction of the sum of the percent of drug retained on the skin surface and drug permeated through the skin from the initial amount of drug used in the donor cell, taken as one-hundred percent.

### Data analysis

The statistical analysis of the data was carried out using Design Expert, version 7.1.5. The data were expressed as the means of three measurements ± SD. The ANOVA also was performed to compare the means (± SD), where applicable. The *P*<0.05 was considered statistically significant.

## RESULTS

### HPLC determination of NAG

**Fig. 2:**
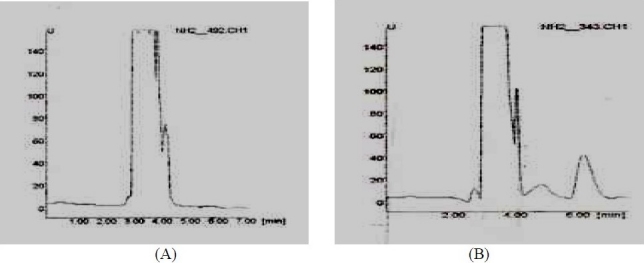
Representative HPLC chromatograms for NAG: (A) blank ethanol 96% solution; (B) NAG spike in ethanol 96% medium (10 µg/ml).

The HPLC method used produced a symmetric peak for NAG, with a retention time of 6.2 min (Fig. 2). The method selectivity was demonstrated on drug-free niosomes, drug-loaded vesicles and ethanolic drug solution; the chromatograms were found to be without interfering peaks. A system suitability test was performed on the results obtained in several representative chromatograms.

The standard curve of NAG solutions was found to be linear in concentrations ranging from 1 to10 µg/ml (y = 115.64 × + 1.2462, r^2^ = 0.9998, with the intercept values not significantly different from zero). The LOQ of this method was 1 µg/ml with the corresponding relative standard deviation of 0.2. The LOD was 0.5 µg/ml at a signal-to-noise ratio of >3.

The inter-day and intra-day variability study results, performed on 6 NAG concentrations (5 times each) are given in Tables 3 and 4. The intra-day precision of assay for quantification of NAG gave coefficient of variation (CV%) lower than 11.27%. The method validation results demonstrated good accuracy, precision, linearity, range and specificity of the method developed.

**Table 3:** Inter-day assay precision and accuracy of the HPLC method for the determination of NAG*

Nominal concentration (µg/ml)	Calculated concentration (µg/ml)	Precision RSD (%)	Accuracy error
1	0.935	6.46	2.41
2	2.009	3.53	-0.454
4	4.07	2.94	-1.925
6	5.93	1.59	1.215
8	8.17	1.01	-2.146
10	9.88	0.42	1.188

* Validation study, n=5 days, five replicates per day

**Table 4:** Intra-day assay precision and accuracy of the HPLC method for the determination of NAG*

Nominal concentration (µg/ml)	Calculated concentration (µg/ml)	Precision RSD (%)	Accuracy error
1	1.02	11.27	2.17
2	2.01	9.55	0.67
4	4.005	7.35	0.07
6	5.96	5.76	0.70
8	7.92	5.82	0.96
10	10.08	6.88	0.80

* Samples were analyzed in five replicates for each concentration per day.

### Vesicle physical characterization

**Fig. 3:**
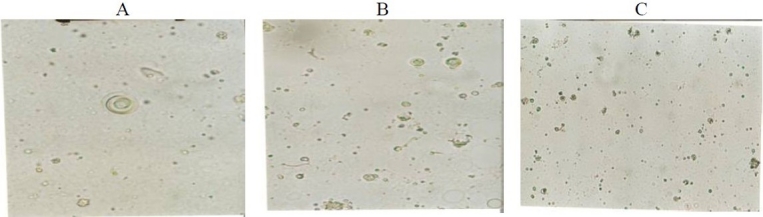
Optical micrographs of NAG niosomes prepared by film hydration method. A) Span 40:chol:DCP (2.8:3.35:1 mole ratio), B) Span 60:chol:DCP (2.8:3.35:1 mole ratio), C) Span 80:chol:DCP (2.8:3.35:1 mole ratio)

**Fig. 4:**
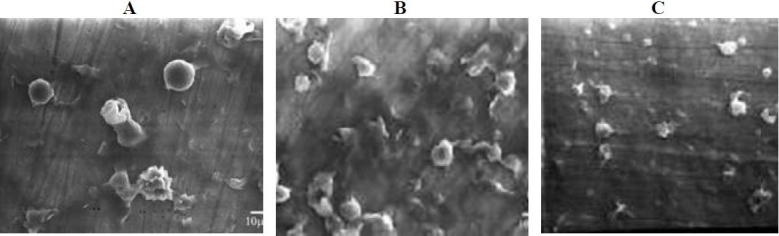
Scanning electron microscopy of NAG prepared by film hydration method. A) Span 40:chol:DCP (2.8:3.35:1 mole ratio), B) Span 60:chol:DCP (2.8:2.35:1 mole ratio), C) Span 80:chol:DCP (2.8.3.35:1 mole ratio).

**Fig. 5:**
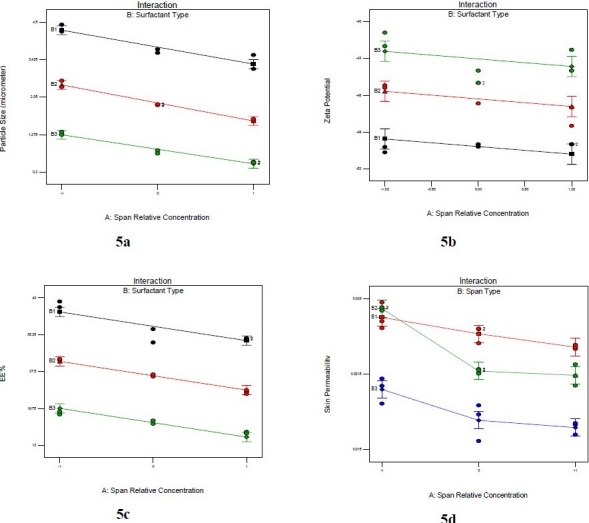
The effects of surfactant type (B1: Span 40, B2: Span 60 and B3: Span 80) and its relative concentration (-1: low or 2.6 mole ratio, 0: medium or 2.9 mole ratio, and 1: high or 3.2 mole ratio) on the particle size (a), zeta potential (b), drug encapsulation efficiency (c), and drug skin permeability (d) of NAG-containing niosomes comprised of Span:chol:DCP (X:3.2:1, mole ratio).

The shape of NAG niosomes was largely spherical to tubular (Fig. 3 and 4). Aggregation was seen to some extent in all formulations. The mean vesicle size of the niosomes varied from about ≤ 0.5 µm to about 4.7 µm (Fig. 5a). The largest vesicle was seen with niosomes made with lower concentrations of Span 40, i.e. relatively higher cholesterol concentrations (Span 40:chol:DCP (2.8:3.35:1 mole ratio). Span 40 formed round shape multilamellar vesicles with less aggregation, while Span 80 formed small multilamellar vesicles and small unilamellar vesicles. Span 60, however, formed niosomes with characteristics between Span 40 and Span 80 vesicles. The negative zeta potential observed with niosomes reflects the presence of negatively-charged DCP on the surface of vesicles (Fig. 5b). However, the absolute values of zeta potential were not dependent on the surfactant relative concentration.

### NAG encapsulation efficiency

The encapsulation efficiency of NAG in niosomes as a function of surfactant type is depicted in Fig. 5c. The statistical analysis of the model for encapsulation efficiency data indicated that Span type and its relative concentration both were significantly effective.

### in vitro release studies

**Fig. 6:**
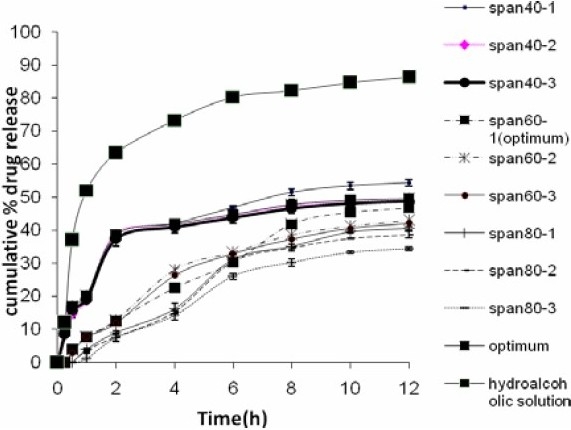
Release profiles of NAG from niosomes into ethanol 96% through a cellulose acetate membrane using Franz diffusion cells, compared to the drug HA solution.

The profiles of *in vitro* release of NAG from different niosomes are shown in Fig. 6. As seen, all noisome formulations released up to about fifty percent of their NAG content over 12 h. Niosomes made with Span 80 had the slowest release rate among all formulations; however, NAG release from the HA solution was significantly faster from all other formulations.

### Rat skin permeation studies

**Fig. 7:**
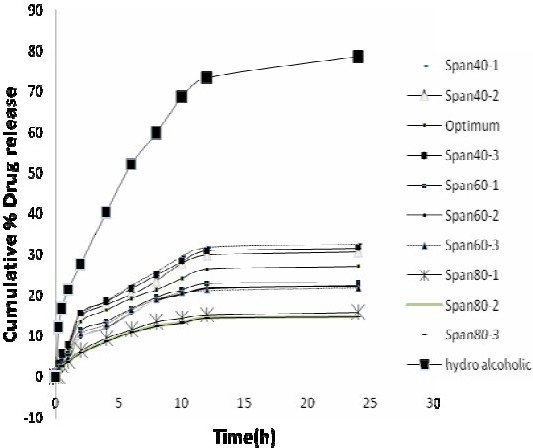
Comparison of drug permeation from NAGcontaining niosomes and HA solution through the rat skin into ethanol 96% at 37 °C. The points represent mean ± SD (n=3).

**Fig. 8:**
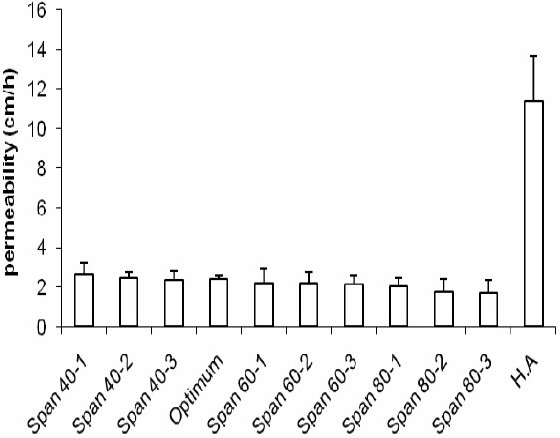
Comparison of drug permeability from NAGcontaining niosomes and HA solution through the rat skin into ethanol 96% at 37 °C.

**Fig. 9:**
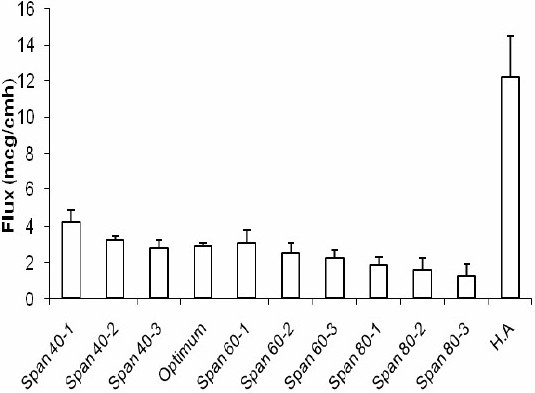
Flux of drug from NAG-containing niosomes and HA solution through the rat skin into ethanol 96% at 37 °C.

**Fig. 10:**
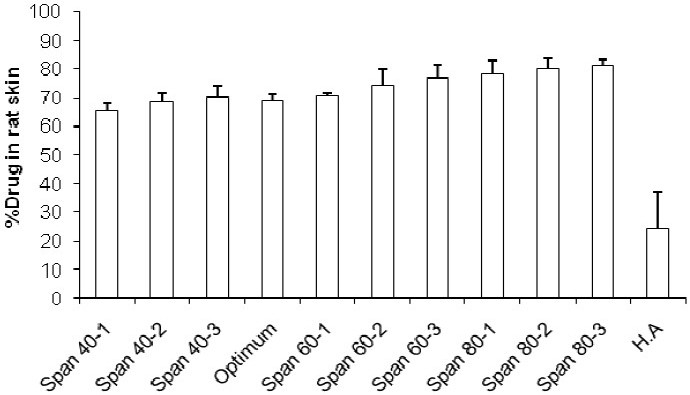
Estimation of residual NAG on the rat skin after 24 h of topical administration of NAG-niosomes and HA solution.

The profiles of NAG permeation through the rat skin following application of NAG containing niosomes and HA solution are shown in Fig. 7. Niosomes made with Span 40, at medium and high relative concentrations, produced higher drug permeation than niosomes made with Span 60 and Span 80 at the same relative concentrations. However, the drug permeation was not significantly different at low concentrations of Span 40 and Span 60. As with the *in vitro* release study, the highest NAG permeation through skin occurred from HA drug solution (Fig. 8 and 9). In contrary, the percent of drug remained on the rat skin (Fig. 10) or estimated to be retained and localized in the skin layers (data not shown), was much greater for NAG-niosomes than hydroalcholic solution. The statistical analysis indicated that skin localization of the drug was not significantly different among noisome formulations.

## DISCUSSION

The smaller size of Span 80 niosomes may be attributed to its lower HLB values, as compared to other Spans having higher HLB values (Fig. 5a.). This was in agreement with findings of Yoshioka et al. (9) who obtained smaller vesicle size by hand shaking method when using surfactants of lower HLB values in the noisome formulation. The smaller size of vesicles at higher relative concentrations of Spans may be explained by the easier formation of the vesicle and/or the better accommodation of the surfactant in the vesicle structure at such condition. The insignificant differences in zeta potentials of various NAG-noisomes seem to be basically related to the presence of almost equal amounts of the negatively-charged component, DCP, in all formulations.

The encapsulation efficiency of NAG in niosomes varied from 15 to 42%. This relatively high entrapment of a very water soluble drug in the lipophilic structure of niosomes, which might be quite unexpected, can be related to the electrostatic association of the oppositely-charged drug molecules and niosomes. The lowest NAG encapsulation efficiency observed with Span 80 niosomes, compared with the highest seen with Span 40 niosomes may be partly explained by the smaller vesicle size of the former, producing smaller aqueous space between vesicle lamellae needed for drug entrapment. Another explanation would be that the smaller size of Span 80 niosomes also provides a larger exposing surface area; thus resulting in greater likelihood of drug leakage from vesicles and lower encapsulation efficiency.

The release and permeation of drug to the receptor was faster where HA drug solution was used in the donor cell, as compared to NAG-niosomes. The drug in solution faced little resistance for its passage from the artificial membrane or the rat skin, in agreement with other reports in the literature(26,27). The relatively higher extent of release and permeation of NAG from Span 40 niosomes than other niosomes was seen as the burst effect in Fig. 6 and Fig. 7. This can be attributed to the higher HLB value of Span40 than other Spans, leading to the easier drug release and permeation from corresponding niosomes, as similarly reported by Vora et al.(10). The higher drug permeability from Span 40 vesicles than other niosomes (Fig. 8 and Fig. 9) can probably be due to the higher fluidity of these vesicles. Span 40 has a relatively lower phase transition temperature (T_c_) and therefore may render more fluidity and leakiness to the corresponding niosomes(26,28). The greater extent of drug release also might be due to the higher amount of drug adsorbed on the outmost layers of Span 40-vesicles. The drug adsorption may similarly explain the higher encapsulation efficiency values obtained with Span 40-niosomes (Fig. 5c.).

The rat skin permeation of NAG was much higher from HA drug solution than NAGniosomes (Fig. 7–9). The drug so permeated can be systemically absorbed, which is undesirable for a topically administered drug. Contrary to the permeation results, the percent of NAG, estimated to be remained or localized in the skin layers, was up to about 6% for various noisome formulations. HA drug solution, however, was only able to retain ≤0.5% of the drug in the skin 24 h following topical administration. This was in agreement with findings of other studies indicating a more sustained drug permeation through skin, and possibly a greater drug deposition into the skin, where drug containing vesicular systems used, as compared to a HA drug solution(26–29).

## CONCLUSION

Niosomes comprising Span:chol:DCP (2.6 to 3.2:3.35:1 mole ratio), were able to entrap relatively high amounts of the very water soluble NAG, partly due to the electrostatic association of oppositely-charged molecules of drug and DCP. The highest drug entrapment was obtained with Span 40 niosomes. The sustained drug release and permeation through the rat skin observed with NAG-niosomes indicated the potentials of the niosomes for effective topical delivery of the drug. This was supported by the higher amount of NAG, estimated to be retained or localized in the skin. In addition, lower systemic absorption, and thus, reduced side effects can be achieved by topical NAG-niosomes.
